# Anxiety and depression in association with morbid obesity: changes with improved physical health after duodenal switch

**DOI:** 10.1186/1477-7525-8-52

**Published:** 2010-05-21

**Authors:** John Roger Andersen, Anny Aasprang, Per Bergsholm, Nils Sletteskog, Villy Våge, Gerd Karin Natvig

**Affiliations:** 1 Faculty of Health Studies, Sogn og Fjordane University College. Box 523, 6803 Førde, Norway; 2 Department of Psychiatry, Førde Central Hospital. 6807 Førde, Norway; 3 Department of Surgery, Førde Central Hospital. 6807 Førde, Norway; 4 Department of Public Health and Primary Health Care, University of Bergen. Box 7804, 5200 Bergen

## Abstract

**Background**

Patients with morbid obesity have an increased risk for anxiety and depression. The "duodenal switch" is perhaps the most effective obesity surgery procedure for inducing weight loss. However, to our knowledge, data on symptoms of anxiety and depression after the duodenal switch are lacking. Furthermore, it has been hypothesized that self-reported physical health is the major predictor of symptoms of depression in patients with morbid obesity. We therefore investigated the symptoms of anxiety and depression before and after the duodenal switch procedure and whether post-operative changes in self-reported physical health were predictive of changes in these symptoms.

**Methods**

Data were assessed before surgery (n = 50), and one (n = 47) and two (n = 44) years afterwards. Symptoms of anxiety and depression were assessed by the "Hospital Anxiety and Depression Scale", and self-reported physical health was assessed by the "Short-Form 36" questionnaire. Linear mixed effect models were used to investigate changes in the symptoms of anxiety and depression. Correlation and linear multiple regression analyses were used to study whether changes in self-reported physical health were predictive of post-operative changes in the symptoms of anxiety and depression.

**Results**

The symptom burden of anxiety and depression were high before surgery but were normalized one and two years afterwards (P < 0.001). The degree of improvement in self-reported physical health was associated with statistically significant reductions in the symptoms of anxiety (P = 0.003) and depression (P = 0.004).

**Conclusions**

The novelty of this study is the large and sustained reductions in the symptoms of anxiety and depression after the duodenal switch procedure, and that these changes were closely associated with improvements in self-reported physical health.

## Introduction

Patients suffering from morbid obesity, have an increased risk for symptoms of anxiety and depression [1]. Interestingly, studies have shown that obesity surgery may lead to significant relief of such symptoms, but also to small improvements or improvements that wane with time [2]. Among obesity surgery procedures, the "duodenal switch" is perhaps the most effective for inducing weight loss [3]. However, to our knowledge, data on symptoms of anxiety and depression after this procedure are lacking. Since different bariatric procedures may give raise to positive effects on health as well as side-effects, it has been argued that each procedure should be carefully documented longitudinally [4,5].

Another interesting issue is the puzzling finding that although the degree of weight loss after obesity surgery may predict changes in symptoms of anxiety and depression, the size of the effect has been rather small [6]. The body mass index (BMI) has also been shown to be a poor predictor of symptoms of depression in patients seeking obesity surgery [7-9]. One theory is that obesity mainly influences mental health through its impact on self-reported physical health, which is defined as physical functioning, physical role functioning, and bodily pain [7]. Some data seems to support this theory, since symptoms of depression have only been shown to be high among patients with morbid obesity who also had poor self-reported physical health, regardless of BMI [7]. Qualitative interviews have also revealed that reduced self-reported physical health is considered to be a substantial burden in patients undergoing obesity surgery [10], Furthermore, a survey showed that depressed patients with morbid obesity were primary motivated by their poor physical health to seek obesity surgery [11]. Although there are no reasons to doubt that reciprocity exists between obesity, self-reported physical health, and depression [12,13], depressive symptomatology has been reported to flow mainly from poor physical health to depression rather than in the reverse direction [14]. In conclusion, there is a lack of knowledge about the predictors of symptoms of anxiety and depression after obesity surgery. Such data may shed more light on how bariatric surgery influences mental health.

Therefore, this study aimed to prospectively assess the symptoms of anxiety and depression in a sample of patients who were treated with the duodenal switch procedure for morbid obesity and to determine whether changes in self-reported physical health was predictive for changes in such symptoms. We hypothesized that anxiety and depression would improve following duodenal switch and that these changes would be related to changes in self-reported physical health.

## Methods

### Patients and study design

The first 51 patients with morbid obesity who were accepted for obesity surgery at Førde Central Hospital were invited to participate in the study. Our bariatric surgery program was initiated in 2001, and the inclusion criteria included BMI ≥ 40.0 or 35.0-39.9 with obesity-related co-morbidities, age 18-60, no alcohol or drug problems, no active psychosis, and failure to lose weight through other methods. Power calculations were performed using a two-sided paired test (predicted effect size = 0.6, providing 90% power, p < 0.05) indicating that at least 32 paired observations would be required to detect changes in the anxiety and depression scores. Data were assessed before surgery (T0), one-year after surgery (T1), and two-years after surgery (T2).

### The treatment: duodenal switch

The duodenal switch (open approach) is performed by resecting the greater curvature of the stomach, leaving a narrow gastric tube of 100 to 120 ml along the lesser curvature. The pylorus is left intact, and the duodenum is divided 3 to 4 cm distal to the pylorus. The small bowel is usually divided 250 cm above the coecum, and the proximal end of the distal small bowel is anastomosed to the proximal end of duodenum (alimentary limb). The distal end of the proximal small bowel is usually anastomosed to the alimentary limb 75 to 100 cm above the coecum (common limb). Due to the malabsorption resulting from the procedure, patients are encouraged to eat a high protein diet and to take prescribed daily doses of vitamins and minerals.

### Demographic characteristics and clinical data

Data were obtained using a standardized form. The patients' age, gender, marital status, employment status, and educational level were noted. A history of anxiety or depression was considered to be present if the patient's general physician confirmed the diagnosis and the patient was on documented treatment. Body weight was measured in light clothing without shoes to the nearest 0.1 kg. Height was measured in a standing position without shoes to the nearest 0.01 m. BMI was calculated as weight divided by height squared (kg/m^2^).

### Symptoms of anxiety and depression

Information on symptoms of anxiety and depression were assessed using the "Hospital Anxiety and Depression Scale" (HADS), a self-report questionnaire comprised of 14 items, with seven items assessing anxiety and seven assessing depression [15]. No items related to somatic issues were included, as the questionnaire was designed to assess symptoms of anxiety and depression in the physically ill. The items were scored on a four-point scale from zero (not present) to three (considerable). The item scores were added, giving sub-scale scores on the anxiety scale and the depression scale from zero to 21. A lower score represented better mental health. The HADS has shown good case-finding properties in primary care and hospital settings for anxiety and depression according to the Diagnostic and Statistical Manual of Mental Disorders and International Classification of Diagnoses. A cut-off score of 8 points on both subscales was found to give an optimal balance between sensitivity and specificity, with both parameters at about 0.80 for depression and anxiety [16]. The HADS has been judged to be well suited for detecting mood disorders among the obese, and have shown good responsiveness to change in patients operated for morbid obesity [6]. Population norm data on the HADS was obtained from the Nord-Trøndelag Health Study (HUNT) in Norway (1995-1997), which was comprised of 57,616 participants aged 20-89 years (53% female)[17].

### Self-reported physical health

Information on self-reported physical health was assessed by the Short Form-36, which is a well-established self-administrated generic measure of the health burden of chronic diseases [18,19]. The Short Form-36 data were used to calculate a physical summary score known as the physical component summary (PCS), which correlates most highly with the subscales for physical functioning, physical role functioning, and bodily pain. Based on conceptual considerations [20], we chose the oblique method to calculate the PCS, which allowed for the correlation of physical and mental health. We also calculated the mental component summary (MCS) according to the same method. The basic version of the SF Health Outcomes™ Scoring Software (Quality Metric Inc. Lincoln, USA) was used to calculate the summary scores.

### Statistics

The patients' HADS data were calculated as means and standard deviations, and as means for the population norm. The number of subjects with HADS scores ≥ 8 points was also assessed. The HADS scores of the population norm were adjusted by age and gender to reflect the same distribution as in our study sample. The method for this adjustment has been described elsewhere [21]. We calculated effect sizes to illustrate the differences in HADS scores between patients and the population norm by subtracting the mean score of the population norm from the mean score of the patient group, divided by the standard deviation of the patient group. Effect sizes were judged against the standard criteria proposed by Cohen [22]: trivial (<0.2), small (0.2 to <0.5), moderate (0.5 to <0.8), and large (≥ 0.8). Mixed-effect models were used to calculate repeated mean changes and 95% CIs for the HADS scores from T0 to T1 and T2. Correlation analyses (Pearson's *r*) and multiple linear regression analyses were used to investigate predictors for changes in the HADS scores from T0 to T2. The choice of variables in the multiple regression analysis was made based on theoretical considerations and previous research [8]. A two tailed p-value of < .05 was considered statistically significant. The mixed linear analyses were conducted with the statistical program R (the R Foundation for Statistical Computing, Vienna, Austria). The remaining analyses were performed using the statistical program SPSS for Windows, version 15.0 (SPSS Inc., Chicago, USA).

### Ethics

This investigation conforms to the principles outlined in the Declaration of Helsinki. The study protocol was approved by the Regional Committee of Ethics in Medicine, West-Norway (registration number: 234.03).

## Results

Informed consent was obtained from all the 51 participants who were invited to participate in the study. However, one patient did not complete the HADS questionnaire at T0 and was excluded from the study. Of the remaining 50 patients, the mean age was 37.9 ± 7.9 years and 56% were women. Other characteristics of the patients are presented in table 1 Forty-seven patients (94%) completed the HADS at T1 and 44 (88%) at T2. The six patients who did not complete the HADS at T2 had very similar characteristics compared to the rest of the sample (data not shown). The HADS anxiety score, HADS depression score, PCS and MCS were significantly correlated with each other at T0 (Table 2) and at T1/T2 (data not shown). Using all available data, the mean Δ BMI from T0 to T2 was -20.0 units; 95% CI, -17.9 to -22.1; P < 0.001. The HADS scores at T0 did not predict changes in BMI after the operation (Ps <0.652). The mean Δ PCS score from T0 to T2 was 21.3 points; 95% CI, 17.6 to 25.0; P < 0.001, and the mean Δ MCS was 12.9 points; 95% CI, 8.1 to 17.7; P < 0.001.

**Table 1:** Patient characteristics (n = 50).

Variables	T0	T1	T2
Body mass index	51.7 ± 7.5	32.7 (5.8)	31.7 (5.7)
Physical component summary	31.9 ± 9.8	52.2 ± 9.5	53.4 ± 8.6
Mental component summary	37.4 ± 12.4	51.8 ± 11.5	50.2 ± 12.3
Married/cohabitation	25 (50.0)	29 (58.0)	25 (50.0)
Education (≥ 13 years)	13 (26.0)	13 (26.0)	13 (26.0)
Employed	27 (54.0)	27 (54.2)	33 (66.0)
On treatment for anxiety	7 (14.0)	6 (12.0)	7 (14.0)
On treatment for depression	12 (24.0)	12 (24.0)	15 (30.0)

Note. T0 is before surgery, T1 is one year after surgery, and T2 is two years after surgery. Age, body mass index, the physical component summary and the mental component summary are presented as means ± standard deviations, while the remaining variables are presented as crude numbers and (percentages). Data were complete for all variables except for the physical component summary and the mental component summary (T2, n = 47 and T2, n = 41).

**Table 2:** Descriptive statistics and correlations among HADS data and the SF-36 summary scores before surgery (N = 50)

	Mean ± SD	HADS-D	PCS	MCS
HADS anxiety	7.8 ± 4.4	0.71 ***	-0.44**	-0.73***
HADS depression	6.3 ± 4.6		-0.49 ***	-0.78 ***
PCS	31.9 ± 9.8			0.53 ***
MCS	37.4 ± 12.4			

Note. HADS: hospital anxiety and depression scale. PCS: physical cumulative summary. MCS: mental cumulative summary. *P < 0.05, ** P < 0.01, *** P < 0.001.

### HADS scores before and after the duodenal switch

**Figure 1:**
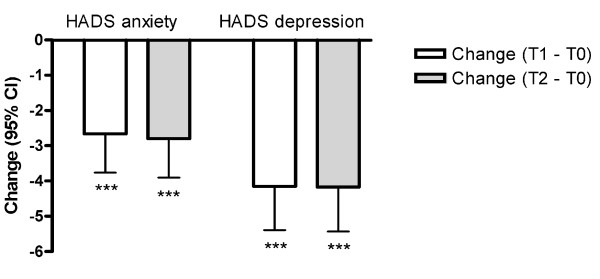
**Mixed effect model analysis: mean changes and 95 CIs in the Hospital Anxiety and Depression Scale (HADS) scores after surgery**. T0 is before surgery, T1 is one year after surgery, and T2 is two years after surgery. *** P < 0.001.

The HADS scores at T0 showed that the patients had considerably more symptoms of anxiety and depression than the population norm (Table 3). The patients' effect sizes at T1 and T2 indicated that their scores had normalized and that the symptoms of depression had improved somewhat more than the symptoms of anxiety (Table 3). The prevalence of HADS scores ≥ 8 points also decreased after surgery. This was reflected by statistically significant changes in the mixed effects analysis (Ps < 0.001) (Figure 1).

**Table 3:** Hospital Anxiety and Depression Scale (HADS) data in the patient group before and after surgery as compared to the population norm.

	T0 (n = 50)	T1 (n = 47)	T2 (n = 44)	Population norm (n = 57616)
*HADS anxiety*				
Mean ± SD	7.8 ± 4.4	5.1 ± 4.0	5.0 ± 3.8	4.4
Effect size	0.77	0.18	0.16	Reference
Score ≥ 8 points (%)	50.0	27.2	22.7	16.1
*HADS depression*				
Mean ± SD	6.3 ± 4.6	2.1 ± 2.3	2.2 ± 3.0	3.0
Effect size	0.72	-0.39	-0.27	Reference
Score ≥ 8 points (%)	36.0	2.1	4.5	8.1

Note. T0 is before surgery, T1 is one year after surgery, and T2 is two years after surgery. The population norm scores are adjusted for age and gender. Effect size is calculated by subtracting the mean score of the population norm from the mean score of the patient group divided by the standard deviation of the patient group. Effect sizes <0.2 are considered trivial compared to the population norm. Effect sizes from 0.2 to <0.5 are considered small, from 0.5 to <0.8 as moderate, and ≥ 0.8 as large. The effects size of the HADS scores can be either zero (identical mean scores in both populations), positive (worse score than the norm population), or negative (better score than the norm population).

However, the number of patients being treated for anxiety and depression were approximately the same at T0 and T2 (Table 1). The prevalence of HADS anxiety scores ≥ 8 points decreased only slightly from 85.7% to 71.4% from T0 to T2 in the patients who were being treated for anxiety before surgery (n = 7). For patients being treated for depression before surgery (n = 12), the prevalence of HADS depression scores ≥ 8 points decreased from 75.0% to 8.3%. Higher HADS scores were associated with being on treatment for anxiety and depression both before and after surgery (Ps < 0.05). The patients being treated for anxiety and depression had poorer PCS scores than the rest of the patients at T0 (Ps < 0.05, but not at T2 (Ps > 0.18).

### Predicting changes in symptoms of anxiety and depression after the duodenal switch

Correlation analyses showed that a higher Δ PCS score was significantly correlated with a greater decrease in the Δ HADS scores, but Δ BMI was not (Table 4). In the multiple linear regression analysis, a higher Δ PCS score was predictive of greater decreases in the Δ HADS scores for anxiety (non-standardized reg. coeff, -0.18; 95% CI, -0.29 to -0.06; P = 0.003) and depression (non-standardized reg. coeff, -0.17; 95% CI, -0.27 to -0.06; P = 0.004) after adjusting for age, gender, and the initial PCS and HADS scores.

**Table 4:** Descriptive statistics and correlations among Δ scores

	Mean ± SD	Δ HADS anxiety	Δ BMI	Δ PCS
Δ HADS anxiety (n = 44)	-3.0 ± 4.0	0.53***	-0.12	-0.45**
Δ HADS depression (n = 44)	-4.5 ± 5.0		0.06	-0.57***
Δ BMI (n = 50)	-20.0 ± 7.5			-0.37*
Δ PCS (n = 41)	21.3 ± 11.7			

Note. HADS: hospital anxiety and depression scale. BMI: body mass index. PCS: physical cumulative summary. The Δ scores were calculated by subtracting the scores assessed two years after surgery from the initial scores.

*P < 0.05, ** P < 0.01, *** P < 0.001.

## Discussion

This is, to our knowledge, the first study to demonstrate a large and sustained reduction in the symptoms of anxiety and depression after the duodenal switch procedure, and that these changes were closely associated with improvements in self-reported physical health. Although this study cannot establish causality, it supports the hypothesis that improved self-reported physical health is a mechanism by which the symptoms of anxiety and depression are decreased in patients undergoing obesity surgery.

Even though studies have shown reductions in symptoms of anxiety and depression after different types of obesity surgery [2,6], the results in the present study are particularly promising. This study adds to the body of data that the duodenal switch is associated with beneficial effects on a range of aspects of health-related quality of life [23-25], despite that one common side-effect after this operation is malodorous flatus [5]. Thus, the side-effects of the duodenal switch do not seem disturbing enough to override the patient's health appraisals. However, the maintenance of an adequate weight loss seems to be crucial for long-term symptom relief [6]. The duodenal switch may therefore be particularly effective, since it has the best long-term weight loss of any obesity operation [3]. However, longer follow-up is required to investigate this issue.

Surprisingly, we found that the number of patients who were being treated for anxiety and depression was quite stable during the study. We can only speculate on the reasons for this finding. The data showed that the patients who were being treated for anxiety before surgery continued to have substantial symptom burdens of anxiety afterwards. However, it is possible that their anxieties were unrelated to obesity to begin with. On the contrary, the patients who were being treated for depression before surgery had very low symptom burdens of depression after surgery. Unfortunately we had no specific information regarding how the general physicians had evaluated the need for continued treatment for depression.

That the symptoms of depression were somewhat more reduced than the symptoms of anxiety is in agreement with some previous work [26,27]. Although symptoms of anxiety often are reduced after obesity surgery, the patients may face challenges related to self-concept, social relations, and skill acquisition [28]. How patients cope with these matters might influence different health outcomes, perhaps anxiety in particular. It has also been reported that some patients may fear regaining their weight [26].

The finding that one point increase in the Δ PCS score was associated with an approximately 0.17 point decrease in the Δ HADS scores can be regarded as clinically significant, since the average Δ PCS score was 21.3 points. However, longitudinal data on this issue is scarce. In one study, improvement in self-reported physical health (the orthogonal physical component summary score of the SF-36) was significantly correlated with a decrease in the "Beck Depression Inventory Score" after lap band surgery [8]. Unfortunately, the change in the self-reported physical health score was not included in the multiple regression analysis in that study.

One particular study seems to contradict the hypothesis that self-reported physical health is a major predictor of anxiety and depression. Improvements in symptoms of depression were documented as early as 2 to 4 weeks after Roux-en-Y gastric bypass, despite the fact that no changes in self-reported physical health had occurred [29]. However, this rapid improvement may have been caused by a realistic hope that a long-term disability was about to be relieved. Thus, we hypothesize that sustained relief of symptoms of depression will only be observed if an improvement in self-reported physical health also occurs.

The finding that that the Δ BMI was not significantly correlated with the Δ HADS scores may be related to the fact that the patients' weight loss could have exceeded a threshold above which few differences in symptoms of anxiety and depression were observed. For example, in the SOS study, the degree of weight loss predicted a greater improvement in the HADS depression score after bariatric surgery [6]. This effect was especially large in patients who lost ≥ 30% of their initial weight, which occurred in 12% of the patients. In our study, 78% of the patients lost ≥ 30% of their initial weight. Thus, our data displayed large effect sizes with little variability.

Strengths of this study are the validity of the HADS and the Short Form-36 and the large changes in these measures after surgery. It is important to note that the HADS questionnaire is not "contaminated" with somatic issues to avoid circular reasoning [15]. There are also clear limitations to the study. First, this was not a randomized controlled trail (RCT). Some of the changes in symptoms of anxiety and depression seen in this study could therefore be partly due to other things than the surgery. However, it has been argued that due to practical and ethical reasons, RCTs are not usually an appropriate standard of evidence for evaluating most surgical treatments, which of necessity must rely on prospective cohort studies [30]. Second, the regression models were quite simple and the sample size was relatively small. Unmeasured variables not included in our analyses could have confounded the results (i.e., binge eating, body image satisfaction etc.). Obesity-related stigma is for instance of particular interest as a predictor for symptoms of depression [31]. However, it is unclear to what degree this perceived stigma is an indicator of depression, a cause, or both. This topic should be further examined in prospective studies. Furthermore, a larger sample size could have allowed us to use structural equation modeling to examine the association between self-reported physical health, anxiety, and depression. Although this method cannot establish causality, it can be a useful tool for testing for reciprocal effects [14]. Finally, we lacked standardized data on anxiety and depression based on a structured clinical interview, as well as an evaluation of the need for continued treatment. Thus, we cannot properly address the finding that no patients were taken off treatment for anxiety and depression despite having better HADS scores.

## Conclusions

In conclusion this study indicated that the duodenal switch was associated with a large reduction in symptoms of anxiety and depression. Although we acknowledge that the causal mechanisms for the observed improvements in symptoms of anxiety and depression can be complex, the main mechanism at play was likely weight loss-induced improvements in self-reported physical health. If this mechanism is correct, it is important to keep in mind when we provide care and treatment to our patients with morbid obesity who suffers from anxiety or depression. Future work should be performed to confirm the findings in this study and investigate other possible mechanisms.

## Competing interests

The authors declare that they have no competing interests.

## Authors' contributions

**JRA **drafted the manuscript and performed the statistical analysis. **AA **helped to draft the manuscript. **PB **participated in the design of the study and helped to draft the manuscript. **NS **participated in the design of the study. **VV **participated in the design of the study and helped to draft the manuscript. **GKN **helped to draft the manuscript. All authors read and approved the final manuscript.
